# Case report: A case of widespread soft tissue infection and multiple abscesses secondary to hidradenitis suppurativa inducing septic shock caused by *Lawsonella clevelandensis* in China

**DOI:** 10.3389/fmed.2024.1392430

**Published:** 2024-08-06

**Authors:** Zhi Lijia, Qi Jia Chao, Li Li, Deng Shikun, Gao Peiyang

**Affiliations:** ^1^Department of Intensive Care Unit, Hospital of Chengdu University of Traditional Chinese Medicine, Chengdu, China; ^2^Department of Respiratory and Critical Care Medicine, Zhangzhou Affiliated Hospital of Fujian Medical University, Zhangzhou, China; ^3^Department of Intensive Care Unit, Chengdu Integrated TCM and Western Medicine Hospital, Chengdu, China; ^4^Department of Intensive Care Unit, Chengdu Pidu District Hospital of Traditional Chinese Medicine, Chengdu, China

**Keywords:** *Lawsonella clevelandensis*, widespread soft tissue infection, hidradenitis suppurativa, multiple abscesses, next generation sequencing

## Abstract

*Lawsonella clevelandensis* is rare to associated with human infection, which may cause abscesses in abdominal cavity, liver, breast, and spine. *Lawsonella clevelandensis* is very difficult to be cultivated in regular manner; detection of 16S rRNA sequence is the main evidence for *L. clevelandensis* infection. The clinical manifestations of *L. clevelandensis* infection resemble other agents of Nocardia, Tuberculosis and non-tuberculous Mycobacterium (NTM) due to their morphologic similarities. Hidradenitis suppurativa (HS) is a chronic inflammatory disorder, which affects the intertriginous skin and is associated with numerous systemic comorbidities. HS eventually leads to severe pain, multiple abscesses, pus discharge, and irreversible tissue destruction. *Lawsonella clevelandensis* has not been reported to cause HS and systemic comorbidities. We presented the case of a 33-year-old male with widespread soft tissue infection and multiple abscesses secondary to HS (Hurley stage III) inducing septic shock caused by *L. clevelandensis* in China. He was diagnosed as HS and treated with intravenous antibiotic empirically. He developed multiple abscesses including lung and scrota. Bacterial and fungal cultures on blood and secretions from multiple skin lesions were all negative. Due to the misdiagnosis and progression of disease, the patient was transferred to intensive care unit, and he underwent drainage of the chest and right hemothorax removal under thoracoscopic. During the hospitalization, the patient developed septic shock and received mechanical ventilation. Computerized tomography (CT) scans revealed mediastinal emphysema, multiple subcutaneous emphysema, and severe pneumonia. Gene analysis of samples of incision and drainage of pus at the skin showed the rare infection of *L. clevelandensis*. Finally, the patients with recurrent soft tissue infections and multiple abscesses with negative microbiological culture results recovered after effective abscess drainage and antibiotic therapy. We suggested that NGS is a crucial supplementary diagnostic tool in individuals with recurrent skin infections and multiple abscesses, especially when conventional diagnostic methods are inconclusive.

## 1 Introduction

*Lawsonella clevelandensis* is an anaerobic and partially acid-fast bacillus, Gram-positive bacillus (1). It is rare to associated with human infection, and it is also regarded as a kind of skin colonization bacteria (2, 3). However, *L. clevelandensis* may cause abscesses in abdominal cavity, liver, breast, spine, lung, and scrotum (1, 4–10).

Meanwhile, *L. clevelandensis* is very difficult to be cultivated in regular manner; detection of 16S rRNA sequence is the main evidence for *L. clevelandensis* infection (6, 7, 10, 11). Interestingly, the clinical manifestations of *L. clevelandensis* infection resemble other agents of Nocardia, Tuberculosis and non-tuberculous Mycobacterium (NTM) due to their morphologic similarities (6, 8, 12, 13). In terms of treatment for *L. clevelandensis*, it was sensitive to most of commonly used antibiotics *in vitro* (14). It was considered as an opportunistic pathogen and might gain extensive drug resistance after treatment of broad-spectrum antibiotics (1, 15). Most of the patients have a good prognosis after thorough surgical intervention with concomitant antimicrobial therapy (12).

Hidradenitis suppurativa (HS), with the estimated prevalence of 1% in most studied countries, is a chronic inflammatory disorder. HS affects the intertriginous skin and is associated with numerous systemic comorbidities. The unrestricted and chronic immune response eventually leads to severe pain, pus discharge, irreversible tissue destruction, contributing to reduced life expectancy. Chronic HS can increase polybacterial colonization, including high enrichment of strictly anaerobic Gram-negative bacteria such as *Prevotella* and *Porphyromonas* spp. as well as *Streptococcus anginosus* (16). To our knowledge, *L. clevelandensis* has not been reported to cause HS and systemic comorbidities. We presented the first case of widespread soft tissue infection and multiple abscesses secondary to HS (Hurley stage III) (5) inducing septic shock caused by *L. clevelandensis* in China.

## 2 Case report

**Figure 1:**
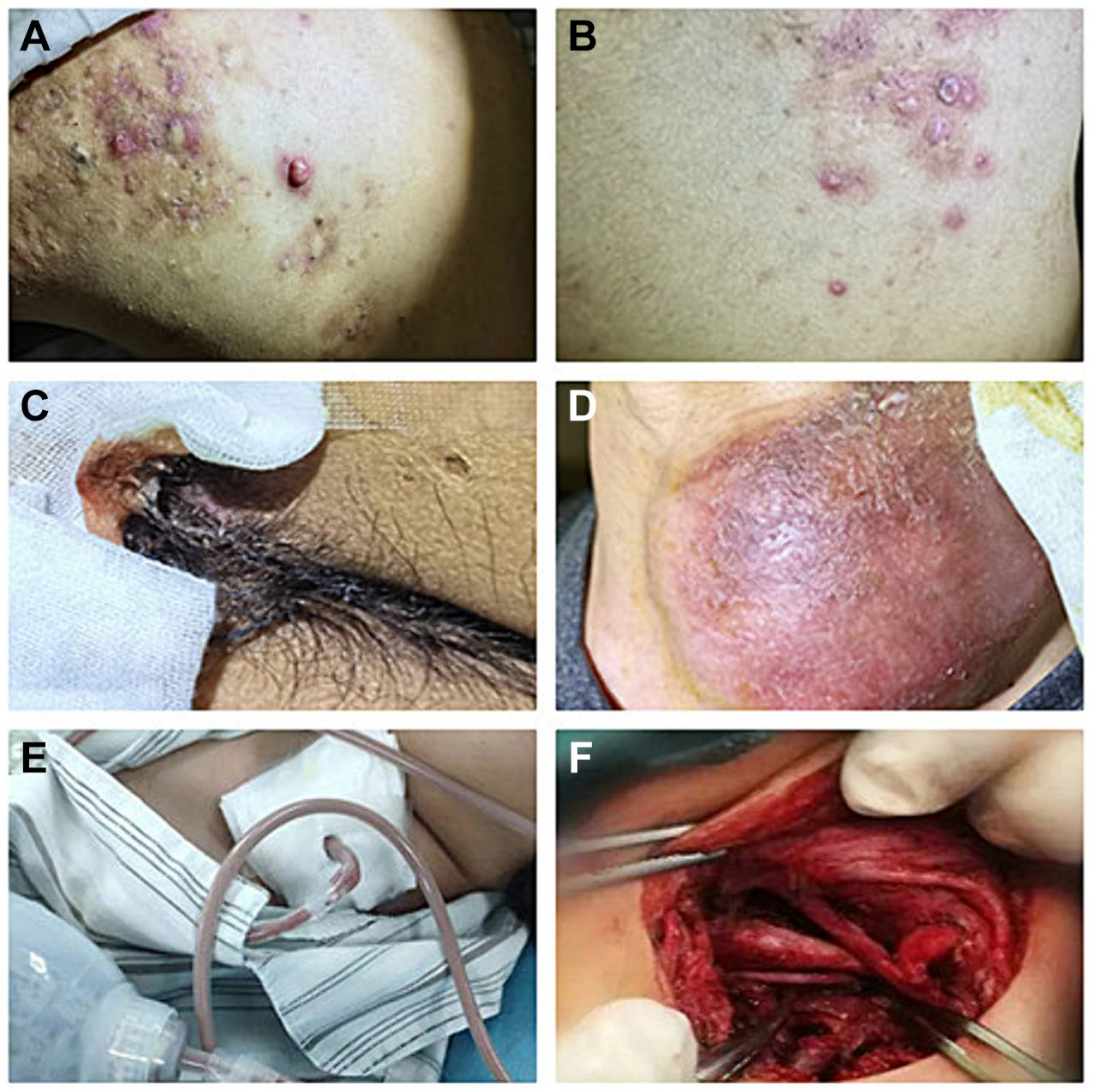
Recurrent rashes and multiple abscesses at admission. **(A)** Recurrent rashes in shoulder; **(B)** recurrent rashes in back. **(C)** Abscesses in scrota; **(D)** abscesses in left neck (8 cm * 7 cm); and **(E,F)** abscess incision drainage.

**Figure 2:**
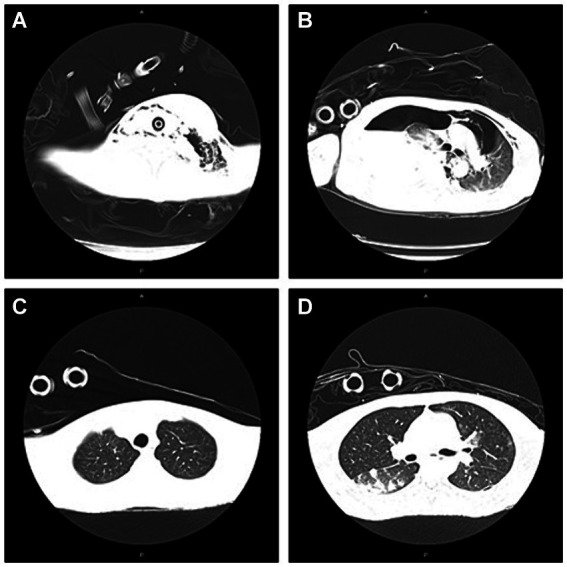
The presentation of CT on neck/chest at admission to ICU and 7th day after adjustment of antibiotic therapy. **(A)** Abscess in the left neck; subcutaneous emphysema in left maxillofacial and neck at admission to ICU; **(B)** Mediastinal emphysema; pneumonia; abscess in the right lung; large amount of fluid pneumothorax on the right side; pneumothorax on the left side at admission to ICU; **(C)** Partial absorption of subcutaneous emphysema in the left maxillofacial, cervical regions mediastinal on Day 21; and **(D)** Partial absorption of emphysema in mediastinal and pneumothorax on Day 21.

A 33-year-old young man with no previous medical history was admitted to our hospital due to recurrent rash on the head and neck, shoulder and back for 2 years, rupture of a left neck mass, and multiple abscesses throughout the body for half a year, which aggravated for 1 month (Figure 1). Continuous discharge of pus lasted after the ulceration of the papules. Before admission, the patient felt that the skin on the left neck is significantly elevated, forming a round soft mass with a diameter of 8 cm, with clear boundaries and a sense of fluctuation (Figure 1). At the same time, the patient complained of skin congestion and swelling in the left inguinal area and scrotum enlargement. After admission, the patient was diagnosed as hidradenitis suppurativa (HS) (Hurley stage III) and treated with intravenous antibiotic empirically (levofloxacin 0.5 g, qd and cefazolin 2 g, q12h). On day 1, the body temperature of patient fluctuated between 37°C and 38.5°C. Dizziness, muscle pain, and other discomforts accompanied with the fever. A total of six pus swab samples were collected for bacterial smear test, acid-fast stain, regular and anaerobic bacterial culture while blood samples for fungal and tuberculosis testing. The specimens on blood and secretions from multiple soft tissue infections were inoculated onto anaerobic blood agar, chocolate and Campylobacter selective agar. Enrichment culture was performed using Robertson’s broth which, at 48 h of incubation, was subcultured onto anaerobic blood agar. All anaerobic blood agar plates were incubated for 10 days, under strict anaerobic conditions (Whitley MG1000 anaerobic work station), at 35°C, in sterile zip-lock bags. No organisms were isolated by means of conventional direct and enrichment culture in a microbiology laboratory (8). Laboratory results were notable for an erythrocyte sedimentation rate (ESR) of 100 mm/h (2–20 mm/h), C-reactive protein (CRP) of 132 mg/L (< 10 mg/L), white blood cell (WBC) 17.5 × 10^9^/L (4–10 × 10^9^/L), procalcitonin (PCT) 0.32 (0–0.05 ng/mL), and IL-6 65.2(0.00–7.00 pg/mL). The parameters of cellular immune were within the normal reference range, and results of TP, HIV were negative. On the fifth day after admission, the patient experienced sudden severe chest pain and respiratory failure (heart rate: 128 beats/min, blood pressure: 87/53 mmHg, respiratory rate: 30 breaths/min, body temperature: 38.6°C). He was transferred to intensive care unit (ICU) for further treatment. Autoimmune antibody spectrum showed antinuclear antibody (+), nuclear particle type 1:100. The antibody test for ANCA-associated vasculitis was negative. It showed rubella virus IgG 19.5 AU/mL, cytomegalovirus IgG 449.1 AU/mL, and herpes simplex virus type 1 IgG 361.5 AU/mL, and total immunoglobulin E: 213 IU/mL. On day 7, computerized tomography (CT) revealed mediastinal emphysema and subcutaneous emphysema in left maxillofacial, neck and chest, multiple lung abscesses and pneumonia, large amount of fluid pneumothorax on the right side, and moderate pneumothorax on the left side (Figure 2). Drainage of the right chest was carried out, and then right hemothorax removal under thoracoscopic was performed urgently. During the hospitalization, the patient developed septic shock. The findings of laboratory tests suggested WBC 25.1 × 10^9^/L (4–10 × 10^9^/L)CRP of 312 mg/L (< 10 mg/L), PCT 6.35 (0–0.05 ng/mL), and IL-6 65.2 (0.00–7.00 pg/mL). And he received active fluid resuscitation by the infusion of norepinephrine and pituitrin to stabilize haemodynamics. Piperacillin/tazobactam (4.5 g, q8h) combined with vancomycin (1 g, q12h) were administered for anti-infection, and invasive mechanical ventilation (PSIMV model, RR: 14/min, oxygen concentration: 40%, ΔP 8 mmHg, PEEP 5 mmHg) was assisted. An incision was made at the left neck, shoulder, back, and scrotum abscess for adequate drainage of pus (Table 1).

**Table 1:** The temperature during hospitalization, the main positive lab indicators, events, and the treatment protocol.

Hospitalization days	Body temperature	Inflammatory indicators	Results	Reference range of normal value	Events	Treatment
Day 1	37.8°C	WBC	11.2	3.5–9.5*10^9^/L	Diagnosed as HS	Levofloxacin 0.5 g qd and cefazolin 2 g q12h
		Count of neutrophil	7.5	1.8–6.3*10^9^/L		
		ESR	100	0–20 mm/H		
		CRP	132	<10 mg/L		
		PCT	0.32	0–0.05 ng/mL		
		IL-6	65.2	0.00–7.00 pg/mL		
Day 5	38.6°C	Antinuclear antibody	++	Negative	Sudden severe chest pain and respiratory distress; transferred to ICU.	
		Auclear particle type	1:100	Negative		
		ANCA-associated vasculitis	Negative	Negative		
		Rubella virus IgG	19.5 AU/mL			
		Cytomegalovirus IgG and	449.1 AU/mL,			
		Herpes simplex virus type 1 IgG	361.5 AU/mL			
		Total IgE	213 IU/mL	<100 IU/mL		
Day 7	39.3°C	WBC	25.1	3.5–9.5*10^9^/L	Septic shock; mediastinal emphysema and subcutaneous emphysema in left maxillofacial, neck and chest, double lower pneumonia, large amount of fluid pneumothorax on the right side and moderate pneumothorax on the left side.	Active fluid resuscitation; iperacillin/tazobactam (4.5 g, q8h) vancomycin (1 g, q12h)
		PCT	6.35	0–0.05 ng/mL		
		IL-6	202.2	0.00–7.00 pg/mL		Active fluid resuscitation; iperacillin/tazobactam (4.5 g, q8h) vancomycin (1 g, q12h)
		CRP	312	<10 mg/L		
Day 12 (5th day after the incision and drainage)	37.7°C	Bacterial and fungal cultures on blood and secretions from multiple skin lesions.	Negative	Negative	Partial absorption of mediastinal emphysema and subcutaneous emphysema (CT).	
		Histopathological examination of the abscess in the left neck.	A large number of neutrophils and tissue necrosis.	Negative		
Day 14	37.8°C	L. clevelandensis infection in the samples of pus from scrotum and neck by NGS.		Negative		Piperacillin sodium/tazobactam sodium (4.5 g, q8h) + minocycline (0.1 g q12h; adequate wound drainage).
Day 21	36.7°C				Hemodynamic and respiratory parameters improved; HFNC	Transferred to the dermatology ward for further treatment.
Discharged	36.3°C				Multiple abscesses throughout the body and recurrent rashes in shoulder and back disappeared after 6 months.	Minocycline for 6 months in outpatient.

HS, Hidradenitis suppuratival; ICU, Intensive care unit; IgE, Immunoglobulin E; CT, Computerized tomography; HFNC, High-flow nasal cannula oxygen therapy; NGS, Next generation sequencing; ESR, Erythrocyte sedimentation rate; ANCA, Anti-neutrophil cytoplasmic antibodies; IgG, Immunoglobulin G; PCT, Procalcitonin; CRP, C-reactive protein; and IL-6, Interleukin-6.

On the 5th day after the thoracic operation, CT showed partial absorption of mediastinal emphysema and subcutaneous emphysema in the left maxillofacial, cervical regions and chest (Figure 2). Histopathological examination of the abscess in the left neck suggested a large number of neutrophils and tissue necrosis. Considering the etiology of the infection is unclear, the patient signed informed consent, and the samples of incision and drainage of pus at the skin of the left neck and scrotal abscess were stored at −20°C (10 mL, respectively), and they were further sent to Genskey Technology Inc. (Shanghai, China) for NGS (No. MBX75011, MBX7453301) within 12 h. DNA was extracted with a TIANamp Micro DNA Kit (DP316, TIANGEN BIOTECH, Beijing, China) following the manufacturer’s instructions. The extracted DNA was fragmented by sonication to yield 200–500 bp fragments. Then, genomic DNA was qualified using a Nanodrop 2000 (Thermo Fisher), and cfDNA fragment distribution was analyzed on a Bioanalyzer 2100 using the High Sensitivity DNA Kit (Agilent Technologies). We quantified all DNA by utilizing the dsDNA HS Assay Kit on a Qubit 3.0 Fluorometer (Life Technologies). The final libraries were sequenced using the BGISEQ-100 platform (Shenzhen, China). Raw data were preprocessed by removing low quality reads, residual adapters, and short reads. Reads that mapped to a human reference genome using Burrows-Wheeler Alignment were removed. Then, the remaining sequencing data were aligned to the databases, which contains 6,350 bacteria, 1,798 DNA viruses, and 1,064 species of fungi to identify the pathogenic sequences. Finally, we deposit nucleotide sequences in the EMBL-EBI databases.^1^

Surprisingly, the results showed that *L. clevelandensis* infection is present, with the abundance being 50.63 and 64.04% in the samples of pus from scrotum and neck, while the sequence numbers are 526 and 894, respectively. Correspondingly, the antibiotics were adjusted to intravenous piperacillin sodium/tazobactam sodium (4.5 g, q8h) plus minocycline (0.1 g, q12h) (15) following an adequate wound drainage on day 14 (scrotum 5–10 mL/each day and neck 20-30 mL/each day). On the 7th day after adjustment of antibiotic therapy (Day 21), hemodynamic and respiratory parameters of the patient were improved. The ventilator and the tracheal intubation were removed and high-flow nasal cannula oxygen therapy (flow 40 L/min, oxygen concentration: 40%) was applied. The patient was transferred to the dermatology ward for further treatment. He was continued on minocycline for 6 months followed in outpatient. Multiple abscesses throughout the body and recurrent rashes in shoulder and back disappeared.

## 3 Discussion

Only a few cases of *L. clevelandensis* related with the formation of abscess were documented worldwide (9–13). Besides, a case series of *L. clevelandensis* vascular graft infections and cardiac infections were reported, which was a serious complication of reconstructive vascular surgery (14). Escapa et al. (7) demonstrated that *L. clevelandensis* is abundantly found in oily skin sites such as the glabella and occiput, which may represent an organism of the human skin and mucosal microbiota. Damage or irritation can presumably lead to soft tissue infection or abscess formation (8).

On the other hand, HS can increase polybacterial colonization, including high enrichment of strictly anaerobic Gram-negative bacteria such as *Prevotella* and *Porphyromonas* spp. as well as *Streptococcus anginosus* (16). The various bacteria residing on the skin as a protective barrier is possibly related to the onset of HS (3). It had been described that severe HS can develop severe septic shock, and the comorbidities of HS include metabolic and cardiovascular disorders, which contribute to reduced life expectancy (6). Besides, a study including over 1,000 patients with septic shock caused by gram-negative pathogens in ICU revealed that inappropriate antibiotic therapy may independently increase nearly 4-fold risk for mortality (1). Necrotizing skin and soft tissue infection accounted for 1.06% of total ICU admissions (2). To our knowledge, it was the first case of widespread soft tissue infection and multiple abscesses secondary to HS inducing septic shock caused by *L. clevelandensis* in China.

Indeed, *L. clevelandensis* is very difficult to be cultivated in regular manner, leading to the relatively high rate of misdiagnosis (15). Detection of 16S rRNA sequence is the main evidence for *L. clevelandensis* infection (10, 11, 17, 18). However, the initial 16S rRNA PCR/sequencing on aspirate fluid yielded negative results sometimes. Data suggested that negative aspirate fluid can be misleading and it should not be used to rule out infection when clinical presentation and radiological findings are strongly suggestive of infection (17). At present, there is no obvious susceptible population to this pathogen infection. The young patient had unhealthy habits including smoking (two packs a day for more than 10 years) and staying up late, we speculated that the immune system dysfunction may be a possible risk for the infection of *L. clevelandensis* in human. NGS is a suitable technique for detection of rare, atypical, and complicated infections, and it simultaneously detect multiple pathogens in one sample. It can provide advantages in the detection of fungi, tuberculosis (TB), viruses, and anaerobic bacteria (19). In the present case, conventional cultures on blood and secretions from multiple soft tissue lesions were all negative, which may lead to the progression of disease to some extent. Prompt application of NGS may be particularly important for persons with weakened immune system (12) or those who are critically ill (20). Finally, NGS analysis for samples of incision and drainage of pus contributed to the diagnosis of *L. clevelandensis*.

There was limited data regarding antibiotic therapy for *L. clevelandensis*. Goldenberger et al. reported that *L. clevelandensis* was sensitive to most antibiotics *in vitro*. However, it was also considered as an opportunistic pathogen that may not be able to avoid acquiring drug-resistance genes after exposure to broad-spectrum antibiotics (15). In terms of the course of treatment, most authors favored prolonged antibiotic therapy, ranging from 2 to 17 months (9, 10). The most common antibiotic was amoxicillin–clavulanate and sulfamethoxazole–trimethoprim, with a minimum treatment duration of 6–8 weeks (8–11). The patient with liver abscess caused by *L. clevelandensis* who had rheumatoid arthritis received 2 weeks of broad-spectrum antibiotics and 4 weeks of monotherapy with oral amoxicillin/clavulanic acid (12). According to the progression of the disease in our case and the different sensitivity to antibiotics for *L. clevelandensis*, we recommended that the treatment strategy should be adjusted based on the growth rate and the response of antibacterial treatment, especially in cases who showed little effect after broad-spectrum and prolonged antibiotics. Importantly, *L. clevelandensis* secondary to HS may lead to widespread soft tissue infections accompanied with multiple abscesses including lung, scrotum, and neck, it can induce mediastinal emphysema and empyema, which was a risk of poor prognosis (21). In addition to effective antibiotic therapy, drainage is also the key to treatment in critically ill patients. The prognosis of patients with *L. clevelandensis* infection are generally favorable, and most of them achieve cure after abscess drainage and prolonged antibiotic therapy (4). Besides, we should realize the limitations of our study that the gold standard of the disease is a wide surgical excision with appropriate flap reconstruction. It may be worthwhile to consider surgical treatment of HS based on the fact that the surgical excision of the HS reduces the recurrence of HS especially for young patients in stabilized general condition (22, 23). In addition, the relatively expensive cost of NGS analysis was also one of the shortcomings in primary medical institutions.

For patients with recurrent soft tissue infections and multiple abscesses with negative microbiological culture results, it should be recommended for gene analysis to identify the pathogens including rare *L. clevelandensis* for refractory wound.

## Data availability statement

The raw data supporting the conclusions of this article will be made available by the authors, without undue reservation.

## Ethics statement

The studies involving humans were approved by Ethics Committee of Hospital of Chengdu University of Traditional Chinese Medicine. The studies were conducted in accordance with the local legislation and institutional requirements. Written informed consent for participation in this study was provided by the participants’ legal guardians/next of kin. Written informed consent was obtained from the individual(s), and minor(s)’ legal guardian/next of kin, for the publication of any potentially identifiable images or data included in this article.

## Author contributions

ZL: Writing – original draft, Writing – review & editing. QJ: Writing – original draft, Writing – review & editing. LL: Conceptualization, Formal Analysis, Investigation, Writing – review & editing. DS: Methodology, Writing – original draft. GP: Conceptualization, Investigation, Writing – original draft, Writing – review & editing.

## Footnotes

^1^https://www.ebi.ac.uk/ena/data/view/PRJNA1110834
